# Evaluation of functional status among patients undergoing maintenance treatments for opioid use disorders

**DOI:** 10.1186/s12954-021-00488-2

**Published:** 2021-04-13

**Authors:** Juan J. Ruíz Ruíz, José M. Martinez Delgado, Nuria García-Marchena, The ANDOPIO Study Group, G. Aguilera Peralta, l. Alaminos, C. Cáceres Jerez, J. L. Navarro González, A. Rodríguez Rodríguez, C. Suárez Márquez, L. Bernal Jimenez, C. Corbalán Guerrero, Y. Crespo Jimenez, J. Diosdado Fernández, S. Girón García, R. Gómez Tortosa, F. J. Jiménez Barea, M. J. Lobo Lara, F. Luque Pérez, M. J. Rodríguez Melgar, J. L. Roquete Castro, J. A. Sánchez Pérez, P. Seijo Ceballos, M. J. Valdayo Boza, F. C. Alcántara López, M. G. Castro Granados, R. Chacón Villafranca, B. De la Fuente Darder, M. Lizaur Barbudo, L. Manchado López, R. Moreno López, A. Moya Mejías, G. M. Castillo Fernández, J. Joya Redondo, G. Jurado De Flores Yepez, C. López Callejas, L. Orozco Carreras, M. Ruiz Martínez, A. M. Sánchez Viñas, M. Álvarez García, I. Bozquez Gómez, C. Conseglieri Ponce, M. D. De Mula Duran, E. Gegundez Arias, J. González Regalado, D. Morales Rojas, J. F. Ramírez López, A. Gil Martínez, F. Herrera Benítez, E. Montanet Fernández, H. Navarro Cabrera, S. Rodríguez Rus, C. Andújar Pérez, F. Bravo López, I. Burgos Bravo, R. Campos Cloute, R. Campos Gómez, R. Founier López, A. Galán Ruiz de la Herranz, P. Gardeta Sabater, F. Gómez Villaespesa Mará, A. Guerrero Florido, F. Luque García, J. M. Martín de la Hinojosa, A. Moreno Arrebola, J. Pretel Pretel, J. Torroba Molina, F. Vázquez García, J. A. Segura Zamudio, B. Baena, E. Cartagena, E. Claro, C. Iglesias Azcue, A. López, A. Morera, P. Osuna, Á. Rodríguez, C. Sánchez, I. Torres, L. Velo, V. Villafuerte, M. M. Vázquez

**Affiliations:** 1 Centro Provincial de Drogodependencias, Málaga, Spain; 2 Centro Provincial de Drogodependencias, Cádiz, Spain; 3 Unidad de Adicciones- Servicio de Medicina Interna, Institut D’Investigació en Ciènces de La Salut Germans Trias I Pujol (IGTP), Campus Can Ruti, Carrer del Canyet s/n, 08916 Badalona, Barcelona Spain; 4 Instituto de Investigación Biomédica de Málaga (IBIMA), Málaga, Spain

**Keywords:** Opioid use disorders, Methadone, Buprenorphine, Functional status, Sexual dysfunction

## Abstract

**Background:**

Methadone and buprenorphine are the most prevalent types of opioid maintenance programs in Andalusia. The main objective is comparing the functional status of patients with pharmacological opioid maintenance treatments according to different socio-demographic characteristic, health and disabilities domains and sexual difficulties.

**Methods:**

A total of 593 patients from the Andalusia community, 329 were undergoing methadone treatment and 264 were undergoing buprenorphine treatment. The patients were interviewed by socio-demographic and opioid-related variables, assessed by functioning, disability and health domains (*WHODAS 2.0.*) and for sexual problems (*PRSexDQ-SALSEX*).

**Results:**

We found significant differences in the socio-demographic and the opioid-related variables as the onset of opioid use, being on previous maintenance programs, opioid intravenous use, the length of previous maintenance programs, polydrug use and elevated seroprevalence rates (HCV and HIV) between the methadone group and the buprenorphine group. Regarding health and disability domains there were differences in the *Understanding and communication* domain, *Getting around* domain, *Participation in society* domain and in the WHODAS 2.0. simple and complex score, favoring buprenorphine-treated patients. The methadone group referred elevated sexual impairments compared with the buprenorphine group. Opioid-related variables as seroprevalence rates, other previous lifetime maintenance program, the daily opioid dosage and the daily alcohol use are the most discriminative variables between both groups. Participation in society variables and sexual problems were the most important clinical variables in distinguishing the methadone group from the buprenorphine group regarding their functional status.

**Conclusions:**

The methadone group showed higher prevalence in opioid dependence-related variables, elevated disabilities in participation in society activities and sexual problems compared with the buprenorphine group. This study shows the importance of carry out a functional evaluation in the healthcare follow-up, especially in those areas related with social activity and with sexual problems.

## Introduction

Opioid dependence is a chronic and relapsing substance use disorder that causes a significant burden on the global community, leading to 9.2 million disabilities per year [1]. In 2016, in Europe, there were 1.3 million opioid users at high risk, of whom only 628,000 were in opioid substitution programs (63% with methadone treatment) [2]. In Spain, the number of admissions for treatment for opioid use disorders showed a decreasing trend since 2010 but remained stable since 2013–2014 [3]. Particularly, in Andalusia (Southern Spain), there were 2842 new cases of treatment requests for opioid dependence in 2017, and only 329 of them were made by women [4]. Currently, the most common pharmacological treatment for these patients is based on methadone or buprenorphine controlled administration to achieve recovery and normalization. Thus, 13,456 patients and 1252 patients benefited from maintenance programs involving methadone and buprenorphine, respectively, in Andalusia [5].

Methadone has been used as the first-line therapy for opioid dependence reducing the risk for heroin use and associated damage for more than fifty years [6, 7]. Methadone is an orally active synthetic full µ-receptor agonist with an inhibitory effect on the NMDA receptor, producing a better analgesic effect and has a longer half-life than does morphine [8]. It is known that methadone treatment should not be stopped abruptly because tolerance and physical dependence are commonly observed. On the other hand, buprenorphine is a partial agonist at the µ-opioid receptors, and its administration results in a lower risk of toxicity [9]. Buprenorphine is used during opioid detoxification for managing withdrawal and reduce cravings with less potential for opioid use than nonprescription full opioid agonists do [10]. The most common side effects of buprenorphine are constipation and nonspecific headache [11].

The maintenance treatments using methadone or buprenorphine have better adherence to treatment for opioid dependence compared with other therapeutic options, such as opioid tapering or psychological therapies alone [12]. Moreover, it is known that maintenance pharmacological treatments were effective in preventing the spread of infectious diseases [13, 14] decreasing violence and the overdose mortality [15, 16], especially when opioids are used with other depressants drugs, such as alcohol and benzodiazepines [17]. Methadone and buprenorphine treatments have been shown to be safe in physical and mental health [18, 19] and have been reported to improve social functioning [18]. Moreover, the effectiveness of the treatment is sensitive and related with other factors as the comorbid use of other substances, the amount of doses used of the opioid and the quality of the therapeutic supporting services [18].

Usually, the length of pharmacological maintenance treatment depends on the patients needs, considering his or her past instability (dysfunction related to work, social relations and behavior) and chronicity (duration of opioid dependence) [11]. The functional status is an important outcome in health care taking into account the ability to participate in activities of daily living including social, cognitive and psychological aspects [20–22]. Moreover, literature has suggested deterioration in health of patients in opioid maintenance treatments [14, 23]. Prolonged opioid use can be associated with clinical debilitating side-effects in patients undergoing maintenance treatment [24]. These patients are likely to suffer from comorbid mood, anxiety, sleep disorders and even other substance use disorders [25–27]. In addition to comorbid disorders related with opioid use previous studies affirmed that sexual dysfunction is frequently associated with opioid use disorders. Age, the presence of comorbid depressive disorders and the long-term use of opioids are other factors related to sexual dysfunctions, mostly in the domain of sexual desire [28–30].

Based on the complexity of the opioid use disorders, it is important to understand this chronic condition across the evaluation of different domains to consider in achieving a good adherence to pharmacological maintenance programs and maximizes the likelihood a long-term recovery [31]. The main objective of this cross-sectional and descriptive study was to compare patients in methadone and buprenorphine maintenance programs in Andalusia according to their socio-demographic characteristics, level of functioning (positive aspects of the interaction between an individual’s health condition and contextual factors) and sexual problems to assess the functional status of opioid patients, and offer guidance based on the evaluation of the disease and supporting the identification of the needs, treatment adjustments, and measurements of effectiveness for these patients, establishing priorities and allocating resources.

## Methods

### Study design and treatment

The present cross-sectional descriptive study involved a data collection of patients undergoing pharmacological maintenance treatments for opioid use disorders from an intra-community Andalusia multicenter called *Servicios Provinciales de Drogodependencias* over a 3-month period from February to April 2017. Most of the patients were recruited from Malaga 214 (33.2%), and the remaining patients were recruited from other Andalusian provinces: 88 (13.7%) patients were recruited from Cadiz, 82 (12.7%) from Seville, 65 (10.1%) from Granada, 63 (9.8%) from Almeria, 46 (7.1%) from Jaen, 55 (8.5%) from Cordoba and 31 (4.8%) from Huelva. We compared both groups using a consecutive sampling technique.

Based on the attendance indicators, approximately 14,000 patients were in opioid maintenance treatment programs in Andalusia during 2016 [5]. Using a bilateral asymptotic 95% confidence interval to determine the sample size and to achieve an accuracy of 0.4%, we determined that we needed at least 576 participants. To compare the effect of the two main pharmacological maintenance treatments in opioid use disorders, we selected patients with opioids use disorders in active pharmacological treatments with methadone or buprenorphine. Therefore, 644 patients were informed to participate in the study and 631 were recruited and signed for consent. Finally, 593 volunteers were selected due to the inclusion criteria, 329 patients undergoing methadone treatment and 264 patients undergoing buprenorphine treatment. The inclusion criteria were being older than 18 years old and undergoing treatment with maintenance medication with methadone or buprenorphine for opioid use disorders with a stable dose for least 90 days. The exclusion were the presence of cognitive impairment and pregnancy.

### Clinical assessments

Study participants were evaluated by trained interviewers and dependence on opioids was confirmed according to the DSM-IV-TR criteria [32]. The participants were assessed using three parameters: (1) *Ficha de Información Básica de Admisión al Tratamiento* (FIBAT), a standardized database of socio-demographic and opioid-related characteristics; (2) The *World Health Disability Assessment Schedule* (WHODAS 2.0) collecting symptoms experienced indicating health problems; and (3) The *Psychotropic-related Sexual Dysfunction Questionnaire* (PRSexDQ-SALSEX) to evaluate sexual problems during the opioid maintenance treatment programs.

The FIBAT database is a computerized record composed by a basic information sheet used for admission to substance treatment programs, including previous medical treatment, education level, employment, lifetime opioid use and variables related to opioid lifetime use, the frequency and quantity of drug consumption, and information about previous treatments.

The WHODAS 2.0. is an instrument developed by the World Health Organization [33] according to the International Classification of Functioning, Disability and Health (ICF) [34] used to quantify different disability domains, as a multidimensional interaction between environmental and personal factors. According to this, disability is a comprehensive term that includes deficiencies, limitations in activity and restrictions in participation to measure the impact of the disorder on daily activities and heath [33]. According to this, his instrument included the study of the limitations and restrictions in participations to measure the impact of a given intervention in different populations in clinical contexts [35]. This instrument displayed good metric properties in clinic and rehabilitation samples [36] and the Spanish version was validated [37]. In the present study, the scores were categorized as follow: none, mild/moderate, or severe/extreme.

The PRSexDQ-SALSEX [38] is a brief sexual dysfunction questionnaire that includes seven questions, with scores range from 0 to 15. The Cronbach’s alpha for the questionnaire was 0.68 in a schizophrenia population and 0.98 in depressive patients [39].

### Statistical analysis

All data in the tables are expressed as percentage of subjects (%) or the mean and standard deviation [mean (SD)] and the differences that had a *p* value of less than 0.05 were considered significant. The statistical significance of the differences in the categorical and normally distributed continuous variables was determined using Fisher’s exact test (chi-squared test).

Finally, a binary logistic regression model was employed to distinguish between methadone- and buprenorphine-treated patients, and the model included all the relevant and significant health and physiological variables related with the opioid maintenance treatments. The goodness of fit for the model was tested with the Hosmer–Lemeshow test. The statistical analyses were carried out with IBM SPSS Statistical version 22 (IBM, Armonk, NY, USA).

## Results

### Socio-demographic characteristics and opioid dependence-related variables

The average opioid-dependent patient was a 47-year-old man (84.3%) with an elementary education (65.7%) who lived with his family (75.4%). There were elevated employment rates among the participants (66.1%) and the 63% had a driving license. The average of opioid onset was 22.6 years and the 57.6% had participated in other maintenance programs or had previously received health services before the last year.

Table 1 describes the socio-demographic and opioid-related variables for participants of the study. There were significant differences in socio-demographic and opioid-related variables between the methadone group and the buprenorphine group. The mean age of the buprenorphine group was younger than methadone group (45.7 years vs. 47.8) and they started opioid dependence later than patients with methadone (21.6 years vs. 23.9 years). The buprenorphine group had a higher educational attainment, elevated employment rates (73.9%), and more prevalence of driving license (71.6%). Regarding the abuse of other substances, there were significant differences found in the daily use in smoked heroin with cocaine (3.6% methadone vs. 0.8% buprenorphine), alcohol (23.4% methadone vs. 17.0% buprenorphine) and non-prescribed benzodiazepines (22.8% methadone vs. 14.4% buprenorphine). Moreover, we found elevated rates of seroprevalences with higher daily opioid dosage in the methadone group.

**Table 1:** Socio-demographic characteristics between the sample groups

Variables	Methadone (*N* = 329)	Buprenorphine (*N* = 264)	*p* value
Sex (%)			
Men	86.3	81.8	0.083
Age [mean ± SD]			
Years	47.8 ± 7.9	45.7 ± 8.4	*0.002*^a^
Educational attainment (%)			
None	1.50	0.4	*0.019*
Elementary	68.3	62.4	
Secondary	27.4	29.7	
University	2.7	7.6	
Occupation (%)			
Employed	59.9	73.9	< *0.001*
Driving license (%)	56.1	71.6	< *0.001*
Cohabiting (%)			
Alone	17.9	17.4	0.157
With family	74.5	76.5	
With friends	2.1	3.8	
Other	5.5	2.3	
Age onset opioid use [mean ± SD] Years	21.6 ± 7.0	23.9 ± 9.6	*0.001*^a^
I.v. opioid (%)	5.2	6.8	< *0.001*
Seroprevalence (%)			
HBV	18.6	9.8	*0.005*
HCV	43.3	25.1	< *0.001*
VIH	13.9	3.9	< *0.001*
TB	2.2	2.0	0.629
Syphilis	0.6	–	–
Previous maintenance program (%)	59.3	74.2	< *0.001*
Length maintenance program (%)			
Less than a year	14.7	32.0	< *0.001*
Between 1 and 3 years	25.0	45.3	
More than 3 years	60.3	22.7	
Daily opioid dosage (mg)			
Low^b^	37.5	24.6	*0.002*
Moderate^c^	48.4	63.4	
High^d^	14.2	12.1	
Substances (in the last 30 days) (%)			
Heroin	2.5	3.5	0.308
Heroin + cocaine	14.8	13.0	0.313
Cocaine	12.9	10.3	0.207
Nicotine	83.5	81.8	0.277
Cannabis	33.5	30.4	0.238
Alcohol	42.1	35.5	0.064
Benzodiazepines	28.3	19	*0.006*
Substances (every day) (%)			
Heroin	0.3	0.4	0.693
Heroin + cocaine	3.6	0.8	*0.017*
Cocaine	3.0	1.9	0.270
Nicotine	76.9	75	0.329
Cannabis	16.7	19.7	0.203
Alcohol	23.4	17.0	*0.035*
Benzodiazepines	22.8	14.4	*0.006*
Current psychiatric medication (%)			
Benzodiazepines	41.6	36.4	0.111
Antidepressants	21.3	22.0	0.458
Antipsychotics	6.4	9.5	0.107
Anticonvulsants	7.9	6.4	0.302
Others	5.8	4.5	0.317

I.v. = Intravenous administration of opioids; HIV = human immunodeficiency virus; HBV = hepatitis B virus; HCV = hepatitis C virus; TB = tuberculosis

^a^*p* value from Student’s *t* test

^b^Low doses: methadone (2.5–27.50 mg), buprenorphine (1–3 mg)

^c^Moderate doses: methadone (30–75 mg), buprenorphine (4–8 mg)

^d^High doses: methadone (80–160 mg), buprenorphine (10–24 mg). *p* values were calculated with Fisher’s exact test or chi-squared test

### Functioning, disabilities and health

The differences between both maintenance treatments in each domain based on WHODAS 2.0 scores are described in Table 2. Regarding disabilities, it should be noted that for the different items the most prevalent answer was *none difficulty* although the methadone group is the one that turns out to have more mild/moderate difficulties in the different responses.

**Table 2:** Differences in disabilities and health domains between the sample groups

WHODAS 2.0 variables	Methadone (*N* = 329)			Buprenorphine (*N* = 264)			*p* value
	None	Mild/moderate	Severe/extreme	None	Mild/moderate	Severe/extreme	
D1.1 Concentrating on performing a task for 10 min (%)	56.4	35.4	8.2	65.7	30.2	4.2	*0.029*
D1.2 Remembering to do important things (%)	43.3	48.5	8.2	53.2	41.1	5.7	*0.046*
D1.3 Analyzing and finding solutions to problems in day-to-day life (%)	47.6	44.8	7.6	55.5	39.6	4.9	0.110
D1.4 Learning a new task (%)	65.9	30.5	3.7	76.2	21.1	2.6	*0.023*
D1.5 Generally understanding what people say (%)	70.1	28.4	1.5	84.5	13.6	1.9	< *0.001*
D1.6 Starting and maintaining a conversation (%)	72.0	25.3	2.7	77.7	20.0	2.3	0.274
Understanding and communication (UAC) (Mean ± SD)	18.6 ± 18.9			13.5 ± 16.3			< *0.001*^a^
D2.1 Standing for long periods, such 30 min (%)	72.9	21.6	5.5	80.4	17.7	1.9	*0.029*
D2.2 Standing up from a sitting position (%)	75.9	22.3	1.8	80.8	17.7	1.5	0.366
D2.3 Moving around inside your home (%)	83.2	15.2	1.5	88.7	11.3	–	*0.045*
D2.4 Getting out of your home (%)	77.4	19.5	3.0	81.9	15.8	2.3	0.407
D2.5 Walking a long distance, such as a kilometer (%)	76.5	18.9	4.6	80.8	15.8	3.4	0.446
Getting around (GAR) (Mean ± SD)	10.7 ± 16.9			7.9 ± 16.0			*0.030*^a^
D3.1 Washing your whole body (%)	83.2	16.2	0.6	87.9	10.2	1.9	*0.043*
D3.2 Getting dressed (%)	87.8	11.9	0.3	90.9	7.5	1.5	0.065
D3.3 Eating (%)	84.1	14.0	1.8	86.4	12.1	1.5	0.741
D3.4 Staying by yourself for a few days (%)	80.8	17.7	1.5	85.7	11.3	3.0	0.052
Self-care (SCA) (Mean ± SD)	7.2 ± 13.3	6.0 ± 14.1	0.309^a^				
D4.1 Dealing with people you do not know (%)	62.8	32.6	4.6	69.8	26.4	3.8	0.201
D4.2 Maintaining a friendship (%)	70.1	25.3	4.6	75.8	20.4	3.8	0.298
D4.3 Getting along with people who are closet to you (%)	82.6	15.9	1.5	83.0	16.6	0.4	0.375
D4.4 Making new friends (%)	59.5	33.8	6.7	66.8	25.7	7.5	0.098
D4.5 Sexual activities (%)	64.6	28.7	6.7	70.6	21.1	8.3	0.103
Getting along with people (GAP) (Mean ± SD)	17.3 ± 20.3			14.8 ± 20.4			0.152^a^
D5.1 Taking care of your household responsibilities (%)	75.0	21.6	3.4	72.7	24.2	3.0	0.747
D5.2 Doing the most important household task well (%)	79.6	17.7	2.7	76.9	21.2	1.9	0.466
D5.3 Getting all of the household work done that you needed to do [N (%)]	70.4	26.5	3.0	71.2	26.1	2.7	0.951
D5.4 Getting your household work done as quickly as needed [N (%)]	64.0	33.5	2.4	69.7	26.9	3.4	0.192
D5.5 Your day-to-day work/school (%)	77.0	22.4	0.5	76.0	19.9	4.1	0.056
D5.6 Doing your most important work/school tasks well (%)	82.1	16.3	1.5	77.6	19.4	3.1	0.412
D5.7 Getting all of the work done that you need to do (%)	78.6	20.4	1.0	78.6	18.4	3.1	0.331
D5.8 Getting your work done as quickly as needed (%)	73.0	25.5	1.5	73.0	24.0	3.1	0.579
Life activities (LAC) (Mean ± SD)	13.1 ± 20.1			13.2 ± 21.0			0.981^a^
D6.1 Problems joining community activities (%)	56.4	34.8	8.8	66.3	23.5	10.2	*0.012*
D6.2 Problems because of barriers or hindrances around you (%)	51.2	40.5	8.2	66.3	26.9	6.8	*0.001*
D6.3 Problems living with dignity because of the attitudes/actions of others (%)	53.0	34.8	12.2	62.9	30.3	6.8	*0.022*
D6.4 Time spent on one’s health condition or its consequences (%)	39.9	52.4	7.6	50.4	45.1	4.5	*0.025*
D6.5 Emotionally affected by your health condition (%)	36.0	44.7	18.3	43.9	43.6	12.5	0.060
D6.6 Your heath is a drain on your financial resources (%)	44.8	34.5	20.7	53.4	31.1	15.5	0.087
D6.7 Family problems because of your health problems (%)	43.0	36.3	20.7	54.5	32.2	13.3	*0.009*
D6.8 Problems performing activities by yourself for relaxation or pleasure (%)	52.7	41.5	5.8	57.2	35.2	7.6	0.258
Participation in society (PSO) (Mean ± SD)	28.0 ± 22.3			22.1 ± 21.5			*0.001*^a^
WHODAS 2.0 simple (Mean ± SD)	54.1 ± 17.3			51.2 ± 17.7			*0.006*^a^
WHODAS 2.0 complex (Mean ± SD)	17.1 ± 14.9			13.8 ± 14.4			*0.049*^a^

*p* values were calculated with Fisher’s exact test or chi-squared test

*WHODAS* World Health Disability Assessment Schedule

^a^*p* value from Student’s *t* test

Respect the *Understanding and communication* domain (UAC), the methadone group responses with higher difficulty than the buprenorphine group in most of the items. In the *Getting around* domain (GAR) and in *Participation in society* domain (PSO), the methadone group responses with higher difficulties than the buprenorphine group. Moreover, we did not found differences between the groups in *Self-care* domain, in *Getting along with people* domain neither in *Life activities* domain.

Overall, the methadone group showed higher WHODAS 2.0. simple and complex score than the buprenorphine group (*p* < 0.010 and *p* < 0.05, respectively). Despite occupation was a differential socio-demographic variable between both groups; we did not found significant differences between employed and unemployed patients in the pharmacological maintenance groups.

### Sexual dysfunction

The PRSexDQ-SALSEX was used to explore the existence of sexual dysfunction in the sample. There were significant differences in the predicted responses between the methadone and the buprenorphine groups: (a) the presence of sexual dysfunction after pharmacological treatment (41.8% vs. 27.2%, respectively) and (b) a sexual alteration spontaneously mentioned to the clinician (43.9% vs. 33.4%, respectively).

Table 3 shows the results obtained by the PRSexDQ-SALSEX between the methadone and buprenorphine groups. The proportion of patients suffering any sexual problem was significantly higher in the methadone group with special attention on those answers showing moderate or severe/extreme difficulties.

**Table 3:** Differences in sexual dysfunction between the sample groups

PRSexDQ-SALSEX variables	Methadone (*N* = 329)				Buprenorphine (*N* = 264)				*p* value
	None	Mild	Moderate	Severe/extreme	None	Mild	Moderate	Severe/extreme	
1. Decrease in desire for sexual activity (%)	51.5	23.8	14.6	10.1	64.2	21.9	6.4	7.5	*0.002*
2. Delay in ejaculation/orgasm (%)	56.4	21.3	13.4	8.8	69.8	15.8	7.9	6.4	*0.008*
3. Unable to ejaculate/to have an orgasm once you begin sexual relation (%)	61.6	26.2	7.6	4.6	76.6	14.3	4.5	4.5	*0.001*
4. Experienced difficulties obtaining an erection/vaginal lubrication once the sexual activity is initiated (%)	60.4	23.8	9.5	6.4	69.4	22.1	5.3	3.0	*0.032*
5. Tolerated changes in sexual relations (%)	39.3	31.4	23.5	5.8	53.2	31.7	12.5	2.6	< *0.001*

*p* values were calculated ANOVA test

PRSexDQ-SALSEX = Psychotropic-related Sexual Dysfunction Questionnaire

### Variables related to the functional status

In order to investigate the most relevant variables and to discriminate patients between the methadone and the buprenorphine groups with variables related with the opioid use, disability domains and sexual dysfunction, a logistic regression analysis was performed including those variables that were different between both groups in the previous evaluation. The logistic regression model is described in Table 4.

**Table 4:** Binary logistic regression analysis for distinguishing the sample groups

Variables	Odd ratio	95% CI		*p* value
		Lower	Upper	
Understanding and communication domain (UAC)	0.990	0.974	1.007	0.242
Getting around domain (GAR)	0.999	0.982	1.017	0.935
Self-care domain (SCA)	1.009	0.990	1.030	0.353
Getting along with people domain (GAP)	1.006	0.993	1.019	0.369
Life activities domain (LA)	1.010	0.996	1.024	0.159
Participation in society domain (PAR)	0.987	0.975	0.999	*0.034*
Sexual dysfunction	1.915	1.247	2.940	*0.003*
Age	0.984	0.959	1.009	0.209
I.v. opioid	1.126	0.542	2.339	0.750
HBV	1.316	0.704	2.462	0.390
HCV	2.056	1.268	3.332	*0.003*
VIH	3.359	1.405	8.033	*0.006*
Previous maintenance program	0.366	0.237	0.565	< *0.001*
Daily opioid dosage	0.438	0.220	0.872	*0.019*
Heroin + Cocaine use every day	6.670	0.742	59.958	0.090
Alcohol use every day	1.682	1.004	2.819	*0.048*
Benzodiazepines use every day	1.298	0.743	2.265	0.359
Constant	0.034			0.019

Variables introduced in the model: UAC domain, GAR domain, SCA domain, GAP domain, LAC domain, PSO domain, sexual dysfunction, age, i.v. opioid, HBV, HCV, VIH, previous maintenance program, daily opioid dosage, heroin + cocaine use everyday, alcohol use everyday and benzodiazepines use everyday

i.v. = intravenous administration of opioid; CI = confidence interval

The most explanatory variables were PSO domain (*p* < 0.034), sexual dysfunction (*p* < 0.003), HCV (*p* < 0.003), VIH (*p* < 0.006), previous lifetime maintenance program (*p* < 0.001), daily opioid dosage (*p* < 0.019), and alcohol use every day (*p* < 0.048). Regarding the odds ratio in the logistic model, the probability of belonging to the buprenorphine group: decreased by 1.3% when PSO domain increases one unit; increases 1.9% when the sexual dysfunction variable increases one unit, increases 2.1% when HCV seroprevalence increases one unit, increases 3.4% when HIV seroprevalence increases one unit, decreased 63% when the previous maintenance treatment variable increases one unit, decreases 56% when the daily doses increased one unit and finally, decreased 1.7% when alcohol use every day increases one unit. PSO domain and sexual dysfunction are the clinical variables most discriminative and regarding the opioid related variables: HCV, HIV, previous opioid maintenance programs, the daily opioid use and the daily alcohol use provide the differential information between the methadone and buprenorphine groups. The Hosmer–Lemeshow test revealed a good fit for the model (*χ*^2^ = 13.669; *p* = 0.091) and the ROC curve showed a good discriminative power (AUC = 0.757) with an optimal cut-point value of 0.1502 (sensitivity of 98% and specificity of 82%).

## Discussion

The preservation of the functional status during the opioid maintenance treatments has to be considered as an important criterion in the selection of pharmacological maintenance programs. The main findings were as follows: (a) There were significant differences in variables related to the opioid use between both groups, suggesting a better social competence for buprenorphine-treated patients; (b) We found differences between both groups in variables related to functioning, disability and health favoring buprenorphine-treated patients; (c) The methadone group had an elevated prevalence on sexual dysfunction than the buprenorphine group; (d) Opioid-related variables as HCV and VIH seroprevalence, previous maintenance program, the daily opioid dosage and the alcohol use were the most discriminative variables between both groups; (e) Participation in society activities and the sexual dysfunction are the most relevant functional variables in distinguishing the methadone group from the buprenorphine group. Our findings suggest a better level of functional capacity of buprenorphine patients compared with methadone patients, however it is possible that other differences underlie these results rather than directly due the opioid medication treatment. Randomized controlled trials are required to explore these differences.

The opioid patient profile in this study is a middle-aged individual employed that uses chronically opioids through smoked administration, older than the samples of young adults described in Spanish studies [40, 41]. However, other characteristics are similar to studies previously reported, including the higher percentage of men (84%) with an elementary education (66%), with family support (the 75.4% lives with family), driving daily (55%) and with other substance use disorders (e.g., nicotine, cannabis and alcohol) [25, 42]. Concerning the substance use, the pattern was similar in both groups with the exception of the daily use of benzodiazepines which was more common in the methadone group, accordingly with the elevated prevalence of benzodiazepine use found among patients in methadone maintenance programs [43].

Evidence revealed a general health and disability impairment described in patients undergoing maintenance treatment [1, 44]. We found higher levels of difficulty in methadone group than the buprenorphine group in those domains related with cognitive variables (i.e., concentration, problem solving, learning and communication); and in those activities related with the agility and personal movement (i.e., standing, moving inside the home, leaving home and walking long distances). Moreover, they showed difficulties regarding participation in society activities, with family issues and social impoverished activities. Neuropsychological studies reported that patients with methadone treatment showed mental impulsivity, less flexibility and difficulties related to verbal working memory tasks [45, 46]. Regarding physical impairment, methadone maintenance patients showed greater difficulty and impaired psychomotor skills in compared with buprenorphine [9]. However, it is important to stress that methadone remains as a safe profile for its use in opiate-addicted patients [7, 47].

Due to the positive correlation found between every health disability domain and sexual dysfunction, modere/severe problems in this area have an impact on the functional status of opioid patients with undergoing maintenance treatment. According, literature reported that opioid patients could experience orgasm dysfunction, a lack of intercourse satisfaction, less sexual desire and a diminished satisfaction after the initiation of methadone treatment [48, 49]. Methadone doses have been related to decreasing orgasms and greater sexual problems compared with buprenorphine treatment [50, 51]. Otherwise, literature is not clear in this regard because some studies justified the existence of comorbid psychiatric problems related with the opioid use affecting to sexual problems [52–54]. Based on their pharmacological action the methadone is likely to produce an intense inhibition of the sexual performance than buprenorphine [55]. Although secondary sexual problems due to buprenorphine treatment have not been well-studied, in a previous study of patients on opioid use disorder treated with buprenorphine, it was reported at least one sexual dysfunction in the 83% of the subjects [56]. Finally, the testosterone replacement therapy could be interesting for those patients with sexual dysfunction [57], although there are described important side effects [58].

These findings described potential differences between pharmacological maintenance treatments, with a better level of functioning of buprenorphine patients compared with methadone patients. Moreover, we are aware about the existence of limitations. First, the participants were not randomized to the different treatment groups and these differences could be related to observed or unobserved confounders. Second, it is conceivable that additional social, comorbid clinical diagnoses and addiction-related variables can influence the functional activity. Third, we could not exclude the impact of the social desirability bias from the measurements used in this study based on the scores taken from the self-report responses. Finally, we cannot exclude the possible influence of the sex in the differences found between groups in the study. Therefore, is required a larger sample size with a balance proportion of men and women in both pharmacological maintenance treatment. The strengths of the study are as follows: the sample size is larger in opioid-dependent patients under maintenance treatment and performed with patients from all the provinces of the Andalusian autonomous community in Spain, a representative region of Southern Europe; and the good metric properties of the clinical questionnaires used.

Although both pharmacological maintenance treatments have been proven as effective treatments, there is a need to carry out harm reduction strategies in opioid use disorders patients with long medical treatments. There is a need to integrate a functional evaluation in the healthcare follow-up, especially in those areas related with social activity and with sexual problems. We consider that an optimal functional interaction with other community members in an important approach to avoid social isolation in order to improve recovery rates.

## Conclusions

In conclusion, these findings suggest that opioid disorder patients with buprenorphine pharmacological maintenance have a better preservation of functional status compared with methadone patients. Opioid-related variables, participation in society activities and the prevalence of sexual dysfunctions are the most discriminative variables between patients undergoing methadone and buprenorphine maintenance treatments. There is a need of integrate a functional and sexual evaluation in the follow-up of opioid pharmacological maintenance treatments due to their impact on treatment adherence.

## Data Availability

Not applicable.
